# Fanconi anemia manifesting as a squamous cell carcinoma of the hard palate: a case report

**DOI:** 10.1186/1746-160X-2-1

**Published:** 2006-01-13

**Authors:** Giulio Gasparini, Gianluigi Longobardi, Roberto Boniello, Alessandro Di Petrillo, Sandro Pelo

**Affiliations:** 1 U.O. Maxillofacial Surgery. Catholic University Medical School, Rome, Italy

## Abstract

Fanconi Anemia is a rare autosomal recessive disorder characterized by various congenital malformations, progressive bone marrow failure at a very young age and of solid tumors development. The authors present a rare case of a squamous cell carcinoma of the hard palate in a Fanconi Anaemia patient. The atypical clinical manifestation rendered the diagnosis more difficult. This case, for age of appearance, sex and localization, is unique in international literature. We recommend a quarterly follow up of the oral-rhino-pharynx complex in FA patients and to consider as carcinomas, all oral lesions that last more than two weeks.

## Background

Fanconi Anemia (FA) is a rare autosomal recessive syndrome (birth incidence of 1 per 350000), first described in 1927 as a progressive lethal anaemia associated with brown pigmentation of skin [1,2]. Subsequently, this term was extended to a syndrome that includes pancytopenia with hypoplastic bone marrow, skeletal, renal and ophtalmological malformations and chromosomal aberrations. The disease involves many organs including skin and genitourinary, musculoskeletal, cardiovascular and neurological systems. The clinical findings in FA patients are hyperpigmentation, small reproductive organs in males, kidney problems, thumbs and arm abnormalities, skeletal anomalies of hip, spine or ribs, low birth weight, short stature, growth retardation, defects of the tissue separating the heart chambers and mental retardation or learning disabilitym [3,4]. Most cases of FA manifest anaemia symptoms during childhood. However, the symptoms may not become apparent until adulthood [5,6]. FA patients are at risk for secondary malignancies, for example leukaemia, squamous cell carcinoma and hepatocellular carcinoma [7-9]. The risk of squamous cell carcinoma development is expecially high in the anogenital region as well as the head and neck region [10] Increased susceptibility of the oral cavity and anogenital region to local predisposing factors, including environmental toxins and viruses [5]. The authors report a new case of hard palate squamous cell carcinoma in a FA patient. The clinical history and localization of the tumour make this case unique.

## Case report

**Figure 1:**
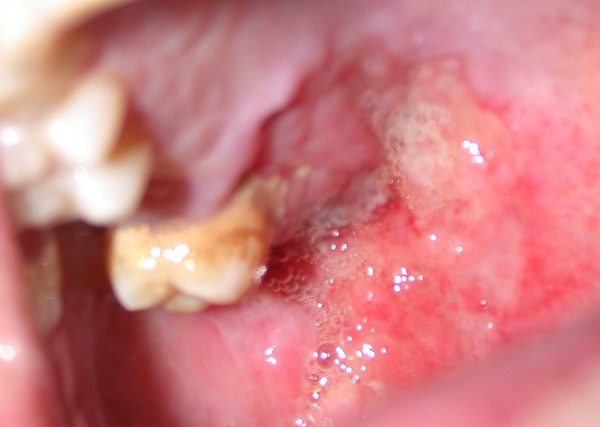
Preoperative hard and soft palate lesion.

The patient, a 27-year-old white male, was referred by a private oral surgeon to our hospital for evaluation of a hard palate lesion that had appeared six months before (Figure 1). The lesion had been diagnosed initialing as gingivitis by the private oral surgeon and treated with local topical medicines without any remission.

At the age of seven the patient was recovered for an orthopaedic trauma. He had been diagnosed with FA at the age of seven after that pancytopenia was noticed during routine blood examinations for orthodontic trauma. He had been treated with androgenic therapy and had not received a bone marrow transplant. The haematological test revealed an early stage of pancytopenia (3,4 × 109/l, Hb 12,3 g/dl, and platelets 13 × 10^9^/l).

**Figure 2:**
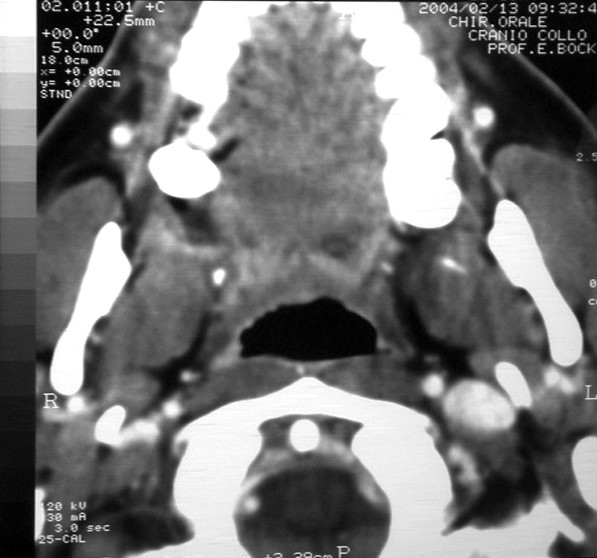
Preoperative CT image.

**Figure 3:**
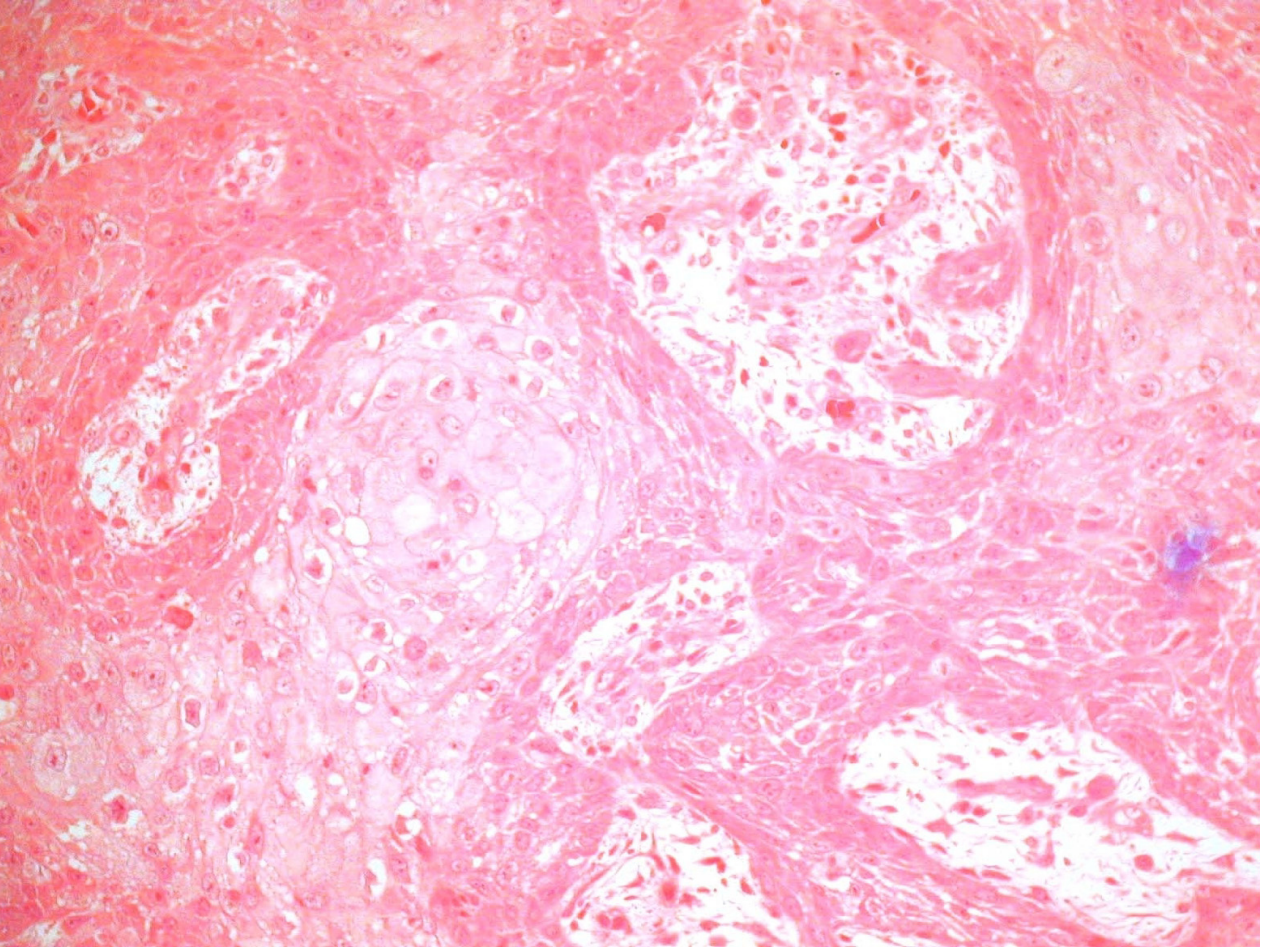
Biopsy finding: Well-differentiated squamous cell carcinoma.

Oral examination revealed a relatively well-defined, nearly circular, concave ulcer measuring 3 × 4 cm, which extended from the hard palatal mucosa in the upper molar region to the adjacent soft palatal mucosa. The surface was erythematous and smooth, with some telangiectasias. Clinical examination showed no regional lymphadenopathy. CT and MR imaging showed a hard and soft tissue mass extending from molar region mucosa to the soft palate mucosa. The nasopharynx appeared normal (Figure 2). No significant cervical lymphadenopathy was seen on the images. An incisional biopsy performed under local anaesthesia revealed a well-differentiated squamous cell carcinoma (Figure 3).

The tumor was surgically removed with a right partial maxillectomy extendiney to homolateral soft mucosa and clear magins. Reconstruction was accomplished with a temporalis muscle flap. The patient has been followed up for 6 months without any evidence of recurrence or metastasis.

## Discussion

Fanconi Anemia is a rare autosomal recessive disorder characterized by various congenital malformations, progressive bone marrow failure at a very young age and of solid tumors development. FA is defined by its cellular hypersensitivity to DNA cross-linking agent such as diepoxybutane (DEB) and mitomycin (MML). Presence of mutations of in one of the different FA genes, FA can be divided into eight complementary groups (A, B, C, D1, D2, E, F, G), with each group having in common the cellular hypersensitivity to cross-linking agent. In the International Fanconi Anemia Registry (IFAR) complementation group A (65%), C (15%) and G (10%) are the most common [11].

The severity is determined by specific complementation group and over all by the type of genetic mutation. Because of these phenotypic differences among complementation groups, FA is a heterogeneous disease. If impaired genetic factors cause an early appearance of the FA syndrome, the same factors may cause an early appearance of malignancies. Thus, there are two distinct groups of patients: (1) severe genetic disturbance with early FA symptoms and early malignancies; (2) mild disturbances with delayed FA symptoms and late malignancies [2]. Kaplan suggested that there are two defects determining the development of cancer in FA patients: defective chromosomal stability and immunodeficiency [12].

Patients that have endured bone marrow transplantation have a greater incidence of malignancies development. In these patients, there are four additional factors including pretransplant total body irradiation, cyclophosphamide treatment, chronic graft versus host disease, and prolonged immunosuppressive treatment after transplantation [2,13,14].

The highest incidence of cancer development in FA patients is reported by Kuttler [11]. In this study he compared the incidence of Hard Neck Squamous Cell Carcinoma (HNSCC) in common population (0.038%) and in FA patients (3%). The first to describe a HNSCC in a FA patient were Esparza and Thompson [4]. Jansisyanont reported that the commonest localizations of squamous cell carcinoma in FA patients in descending order are: tongue, anogenital region, pharynx, larynx, oral mucosa, mandible and skin [13].

Lustig published a review of the international bibliography on the HNSCC in FA patients [2]. He presented 17 cases. In 13 patients the cancer localization was intra-oral. In 9 cases of these 13, the tongue was involved. According with this, in FA patients the tongue cancer incidence is 69%, while in non FA patients the incidence varies by 10 to 16% [2].

Kuttler in 2003 referred that 19 of 754 patients in the International Fanconi Anaemia Registry (3%) had HNSCC [11]. In the same year Bremer presented two cases of HNSCC [15], but in international literature no article has reported a hard palate localization of HNSCC.

The male:female ratio of HNSCC in normal population is 2:1 while Reed asserted the reversed ratio in FA patients [16]. FA patients develop squamous cell carcinoma at significantly earlier age than the general population. Kenedy and Hart reported an average age of 27 years in FA patients [17] and the average time between age of FA diagnosis and cancer development is 10.5 years [2].

The treatment of malignancies in FA patients with HNSCC is similar to the general population with similar pathologies. The aim is the tumour resection oncologic radicality. The main preoperative problem in patients with FA is the associated bone marrow failure, requiring preoperative haematologic consultations. The possibility of blood and platelet transfusion before surgery must be considered. We think that the first approach in FA patients is surgical resection of primary HNSCC with, if necessary, neck dissection and reconstruction. Generally, FA patients withstand surgical procedures very well. A further concern for the surgeon is the development of postoperative complications, including wound infections and haematoma. Although our patient did not develop postoperative complications, FA patients can have serious problems in adjuvant therapy due to increased susceptibility to mutagenic stimuli [2,11,13].

In FA patients, radiotherapy and chemotherapy follow different therapeutic principles. In FA patients the increased susceptibility to XRT and CTx can present problems to determine and to deliver a cancericidal dose without causing significant damage to normal tissue. Thus, in these patients standard doses for adjuvant therapy are generally reduced. Furthermore, the use of conventional protocols, which include cross-linking agents, can cause severe systemic complications, including irreversible aplastic anaemia and catastrophic organ damage [15,18].

Because SCC in FA is difficult to treat once advanced, it is necessary to diagnose malignancies at early stage. We agree with the protocol proposed by Kutler [11]. He suggests a careful biannual screening of the oral cavity and oropharynx that should start between the ages of 15 and 20. However, in patients with FA with histrory of leucoplakia or recurrent oral lesions, head and neck examinations are recommended every six or eight weeks.

## Conclusion

We report a unique localization of hard and soft squamous cell carcinoma in a FA patient. The atypical clinical manifestation rendered the diagnosis more difficult. We recommend a quarterly follow up of the oral-rhino-pharynx complex in FA patients and to consider as carcinomas, all oral lesions that last more than two weeks.

## Competing interests

The author(s) declare that they have no competing interests.

## Authors' contributions

GG drafted the manuscript. GL, RB and ADP carried out the literature search. All authors participated in the treatment of the patient. All authors read and approved the final manuscript.
